# The diversity analysis and gene function prediction of intestinal bacteria in three equine species

**DOI:** 10.3389/fmicb.2022.973828

**Published:** 2022-09-07

**Authors:** Wuyundalai Bao, Jinghe Yu, Yuxing He, Mingchao Liu, Xiaofeng Yang

**Affiliations:** College of Food Science and Engineering, Inner Mongolia Agricultural University, Hohhot, China

**Keywords:** pony, wild ass, zebra, feces, bacterial diversity, analysis

## Abstract

The intestinal flora has a variety of physiological functions involved in the regulation of host metabolism, immunity and endocrinology, and plays an important role in maintaining the health of the host. In this study, we used high-throughput sequencing technology to analyze the intestinal bacterial diversity and their gene functions in three equine species of the genus Shetland Pony (SP), Mongolian Wild Ass (MA), and Plain Zebra (PZ) in captivity in two wildlife parks in Inner Mongolia Autonomous Region, China. The results showed that only the SP intestinal bacterial abundance index (Chao1) was significantly different (*P* < 0.05) between the same species in the two wildlife parks, but neither the intestinal bacterial diversity index (Shannon) nor the community composition were significantly different (*P* > 0.05). The bacterial abundance index (Chao1) was significantly higher in MA than SP (*P* < 0.05) and highly significantly higher than PZ (*P* < 0.01); the bacterial diversity index (Shannon) was higher in MA than PZ, but there was no significant difference, but both MA and PZ were significantly higher than SP (*P* < 0.05). Moreover, the intestinal bacterial community composition was significantly different among the three equine species (*P* = 0.001). The dominant bacterial phyla for SP, MA, and PZ were Firmicutes and Bacteroidota; among them, the bacterial family with the highest relative abundance was Lachnospiraceae and the bacterial genus was *Rikenellaceae_RC9_gut_group*. Analysis of the metabolic gene functions of intestinal bacteria revealed that the highest relative abundance at Pathway level 2 was for global and overview maps; at Pathway level 3, the highest relative abundance was for biosynthesis of secondary metabolites. In sum, the intestinal bacterial community composition and diversity of the above three equine species differed significantly, but their metabolic gene functions were similar. Moreover, the results of this manuscript fill the gap in the study of intestinal bacterial diversity in SP, MA, and PZ. It also provides a reference for the study of the dominant bacteria in the intestinal microorganisms of these three equine species and the discovery of novel functional genes.

## Introduction

The ancestors of the equine genus appeared in North America during the Eocene of the Tertiary. Over the next tens of millions of years, they reached Eurasia several times via the Bering Strait, but left no descendants of extant species. The most recent common ancestor of extant equines occurred during the Pliocene (Jonsson et al., 2014). Equine animals mainly include horses, donkeys, zebras, etc. (Geigl and Grange, 2012). Among them, the pony as one of the equine animals, because of its small and exquisite, intelligent, gentle nature, and popular. It can be used for ornamental, recreational, experimental, and labor purposes, and is also a good “friend” for children and the elderly (Kader et al., 2015). It is especially valuable because of its scarcity, and can be considered the treasure of horses. Secondly, the Mongolian wild ass (MA) (*Equus hemionus kulan*) is mainly distributed in Central Asia and West Asia, and in Inner Mongolia, Gansu, and Xinjiang in China, and is a first-class protected animal in China, International Union for Conservation of Nature (IUCN) endangered level, and is included in convention on international trade in endangered species of wild fauna and flora (CITES) Appendix I (Shao et al., 2020). In addition, the zebra (*Equus zebra*) is an endemic species of Africa (Larison et al., 2021). The zebra’s adaptable digestive system allows it to survive in low-nutrient conditions, making it superior to other herbivores. Zebras are more resistant to African diseases than horses, but they have never been domesticated as domestic animals, nor have they been able to crossbreed with horses and donkeys (Orlando, 2015). The above three species of equine animals are generally found in zoos and visited by people because they are very ornamental and entertaining. Therefore, it is necessary to protect these equines and conduct research on them, such as animal breeding, feeding management, and research on intestinal microorganisms.

It can be also said that the intestinal flora is an important organism in animals (Kobayashi et al., 2021), and the study of animals without intestinal microorganisms can no longer fully reflect the whole life activities of animals. Moreover, equine animals are monogastric animals, and their digestive metabolism and nutrient intake also have certain special features (Fehlberg et al., 2020). Because of this, equids have a unique anatomy and food type. Unlike ruminants, the anterior part of the digestive tract of equids is dominated by enzymatic digestion, similar to monogastric animals; the posterior part of the digestive tract is the hindgut of equids, mainly consisting of the cecum and colon, which is the main site of microbial fermentation, and the microflora of the hindgut turns plant materials into short-chain fatty acids, such as acetate, propionate and butyrate, through fermentation, providing most of the energy required by equids on a daily basis (Biddle et al., 2013). The well-developed caecum of equines is similar to the rumen of ruminants and is dominated by microbial digestion (Graham-Thiers and Bowen, 2011; Harris et al., 2017). The gastrointestinal tract of equids contains a wide variety of microorganisms, including fungi, parasites, protozoa, archaea, viruses, and bacteria (Sekirov et al., 2010; Illiano et al., 2020). Different microbial communities correspond to different physiological functions (Rodriguez-Verdugo, 2021), for example, some are responsible for the digestion of host nutrients (Harris et al., 2017), some undertake the regulation of organismal immunity (Lerner et al., 2016; Vieira et al., 2018), while some act as pathogenic bacteria affecting the health of the host (Hornef, 2015). It has been reported that changes in the structure and diversity of the intestinal flora of equine animals are closely related to the occurrence and development of organismal health and diseases (Dougal et al., 2013), such as Clostridiaceae (Liu et al., 2018), Lachnospiraceae (Surana and Kasper, 2017) Fibrobacteraceae (Gharechahi et al., 2020), and Ruminococcaceae (Welch et al., 2020) are major fiber-degrading bacteria that play an important role in the maintenance of equine health due to their extensive carbohydrate-degrading activity. *Salmonella* and *Colibacillus* can cause diarrhea and sepsis in horses (Xia et al., 2019); Lacetospiraceae and Ruminococcaceae bacteria are reduced in the intestine of horses with diarrhea (Venable et al., 2013); Bacteroidetes and Proteobacteria were increased, Firmicutes and verrucomicrobia were decreased in the intestinal of horses with equine grass disease (Garber et al., 2020), etc. Therefore, an accurate and rapid analysis of the structure and diversity of the intestinal flora is important to resolve the relationship between intestinal flora and disease and to protect the health of the organism. Current research on intestinal flora focuses on the analysis of microorganisms in feces and the determination of the composition of microorganisms in the intestine (Zhu et al., 2021). Study of the intestinal microbiota of equine genera by traditional culture method has certain limitations (Milinovich et al., 2006), as approximately 70% of the microbiota cannot be cultured in the laboratory, making it difficult to determine the entire microbial composition and largely limiting its application in taxonomy (Julliand and Grimm, 2016; Park et al., 2021). The 16S ribosomal RNA (16S rRNA) gene high-throughput sequencing technology, as a novel technical tool for microbial diversity research, is now widely used in animal intestinal flora research, such as Illumina Hiseq-based technology to study different species (Liu et al., 2020), age (Xing et al., 2020), site (Su et al., 2020), and other The structure and diversity of the intestinal microflora under the influence of different factors. In addition, the prediction of intestinal microbial gene function by PICRUSt2 analysis method to further explore the relationship between intestinal flora and host has become a hot research topic.

To the best of our knowledge, no studies have been reported on the intestinal bacterial diversity of Shetland Pony (SP), MA, and Plains Zebra (PZ), and information on the structure, abundance, and gene functions of their intestinal bacteria is unknown. Therefore, this study was devoted to reveal the intestinal bacterial diversity of SP, MA, and PZ (*Equus hippotigris burchellii*), and to predict their bacterial gene functions for the study of the intestinal flora of equine animals.

## Materials and methods

### Fecal sample information and host phylogeny

**FIGURE 1:**
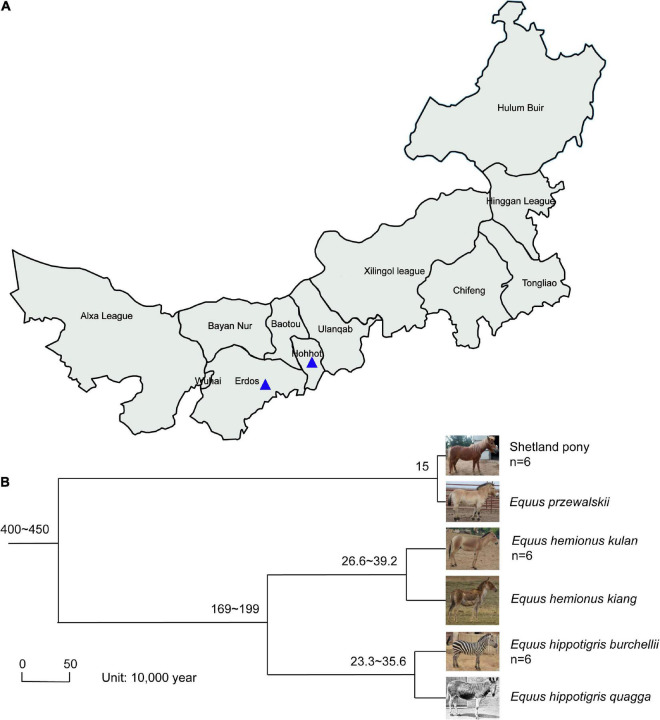
Location of fecal sample collection and host phylogeny plot; Photographs **(A)**; of *Equus hemionus kulan* and *Equus hippotigris quagga* in panel **(B)** are from Baidu search engine. The numbers represent the confidence intervals of the age of differentiation; *n* represents the sample size.

Fecal samples of SP, MA, and PZ were collected from August 25, 2021 to September 5, 2021 at Erdos Wild Animal Park (ES) and Hohhot Daqingshan Wild Animal Park (HT) for the study. The specific collection and delivery methods were as follows: animal feces were broken open with a sterile spoon, and the central part of the feces was clamped as much as possible, immediately loaded into sterile 50 mL centrifuge tubes, then sealed with sealing film and written with the sample information, and stored at an incubator with dry ice; and transported to the laboratory and stored in a −80°C refrigerator for backup, sample collection information is shown in Figure 1A and Supplementary Table 1. The host phylogeny between SP, MA, and PZ is shown in Figure 1B (Waddell et al., 1999; Achilli et al., 2012; Orlando et al., 2013; Vilstrup et al., 2013; Der Sarkissian et al., 2015).

### DNA extraction and polymerase chain reaction amplification of 16S rRNA gene sequences

Total DNA from microbial communities was extracted according to the instructions of E.Z.N.A.^®^ soil DNA kit (Omega Bio-tek, Norcross, GA, United States), and the quality of DNA extraction was detected by 1% agarose gel electrophoresis, and DNA concentration and purity were determined using NanoDrop 2000. The 16S rRNA genes V3–V4 region were amplified using 338F (5′-ACTCCTACGGGAGGCAGCAG-3′) and 806R (5′-GGACTACHVGGGTWTCTAAT-3′). The PCR amplification of the V3–V4 region (Chen et al., 2021) was performed with the following amplification procedure: pre-denaturation at 95°C for 3 min, 27 cycles (denaturation at 95°C for 30 s, annealing at 55°C for 30 s, extension at 72°C for 30 s), followed by stable extension at 72°C for 10 min, and finally storage at 4°C. The PCR reaction system was: 5 × *TransStart FastPfu* buffer 4 μL, 2.5 mM dNTPs 2 μL, upstream primer (5 μM) 0.8 μL, downstream primer (5 μM) 0.8 μL, *TransStart FastPfu* DNA polymerase 0.4 μL, template DNA 10 ng, ddH_2_O to make up to 20 μL. Three replicates per sample.

### Illumina Miseq sequencing of 16S rRNA genes sequences

Polymerase chain reaction products from the same samples were mixed and recovered using 2% agarose gels, and the recovered products were purified using AxyPrep DNA Gel Extraction Kit (Axygen Biosciences, Union City, CA, United States), detected by 2% agarose gel electrophoresis, and detected by Quantus™ Fluorometer (Promega, United States) for quantification of recovered products (Xue et al., 2017). Library construction was performed using NEXTflex™ Rapid DNA-Seq Kit (Bioo Scientific, United States): (1) splice linkage; (2) removal of splice self-linked fragments using magnetic bead screening; (3) enrichment of library template using PCR amplification; (4) recovery of PCR products by magnetic beads to obtain the final library. Sequencing was performed using Illumina’s Miseq PE300/NovaSeq PE250 platform (Majorbio Bio-Pharm Technology Co. Ltd, Shanghai, China). The raw data were uploaded to NCBI Sequence Read Archive (SRA) database (Accession Number: PRJNA848432).

### Quality control of sequencing data

Quality control of the raw sequenced sequences was performed using fastp software^1^ (version 0.20.0) (Chen et al., 2018) and spliced using FLASH software^2^ (version 1.2.7) (Luo et al., 2021) for splicing: filter the bases with quality values below 20 at the end of the reads, set a window of 50 bp, truncate the back-end bases from the window if the average quality value within the window is below 20, filter the reads below 50 bp after quality control (QC), and remove the reads containing N bases; according to the overlap relationship between pair end (PE) reads, splice (merge) pairs of reads into one sequence, the minimum overlap length is 10 bp; the maximum mismatch ratio allowed in the overlap region of spliced sequences is 0.2, and screen the non-conforming sequences; Samples were distinguished based on the barcode and primers at the first and last ends of the sequences, and the sequence orientation was adjusted, with the number of mismatches allowed for barcode being 0 and the maximum number of primer mismatches being 2. Using UPARSE software^3^ (Fakhruddin et al., 2022), the sequences were clustered based on the sequences were Operational Taxonomic Units (OTU) clustered and chimeras were removed based on 97% similarity (Haas et al., 2011). Each sequence was annotated for species classification using RDP classifier software^4^ (Wang et al., 2007), compared to the Silva 16S rRNA database (version 138), and a comparison threshold of 70% was set.

### Data analysis

Shetland Pony, MA, and PZ fecal samples were grouped by sample collection site (ES and HT), and sample sequences were calculated using Mothur software (v.1.30.2), and alpha diversity index analysis was performed on the sample sequences at the OTU level after drawing a flat by minimum sample sequence, i.e., the same species bacterial community abundance index (Chao1) and diversity index (Shannon) using student’s *t*-test to detect whether the index values were significantly different between the two regions, and principal co-ordinates analysis (PCoA) analysis using the R language (version 3.3.1) for plotting and using Adonis to test for differences between groups to synthesize the similarity of bacterial diversity and community composition between the same species in captivity in the two wildlife parks; The same method was used to examine whether the bacterial community abundance index (Chao1) and diversity index (Shannon) were significantly different among the three equine species using student’s *t*-test, and PCoA analysis using the R language (version 3.3.1) for plotting and using Adonis to test for differences between groups. The similarity of bacterial diversity and community composition among the three equine genera were synthesized. The R language (version 3.3.1) tool was used to count and make Venn diagrams, and a sample table with a 97% similarity level at the taxonomic level was chosen for the analysis. The relative abundance of species at each taxonomic level was analyzed using Qiime (version 1.9.1) software, and the Bar diagram of the bacterial community was drawn using the R language tool. Using the R language (version 3.3.1) vegan package, a hierarchical clustering heatmap was drawn for the top 50 genera in relative abundance. For relative abundance of bacterial communities among different species, differences in species were calculated at different taxonomic levels between groups and plotted against the top five species with significantly different relative abundances using the stats package of R software (version 3.3.1), using student’s *t*-test method. Kyoto Encyclopedia of Genes and Genomes (KEGG) Ortholog (KO) information corresponding to OTU was obtained using PICRUSt2 software; and the relative abundance of each KO was calculated. The Pathway information of the three levels was resolved by the KEGG database,^5^ and the relative abundance of each level-related gene was obtained separately. Then one-way analysis of variance (ANOVA) was performed on the Pathway information of Pathway level 2 and Pathway level 3 related functional genes in three equine species to determine the significance of pathway differences in their metabolic gene functions relative to the top five in abundance and map.

## Results and analysis

### Analysis of intestinal bacterial diversity and community composition of the same species in different regions

**FIGURE 2:**
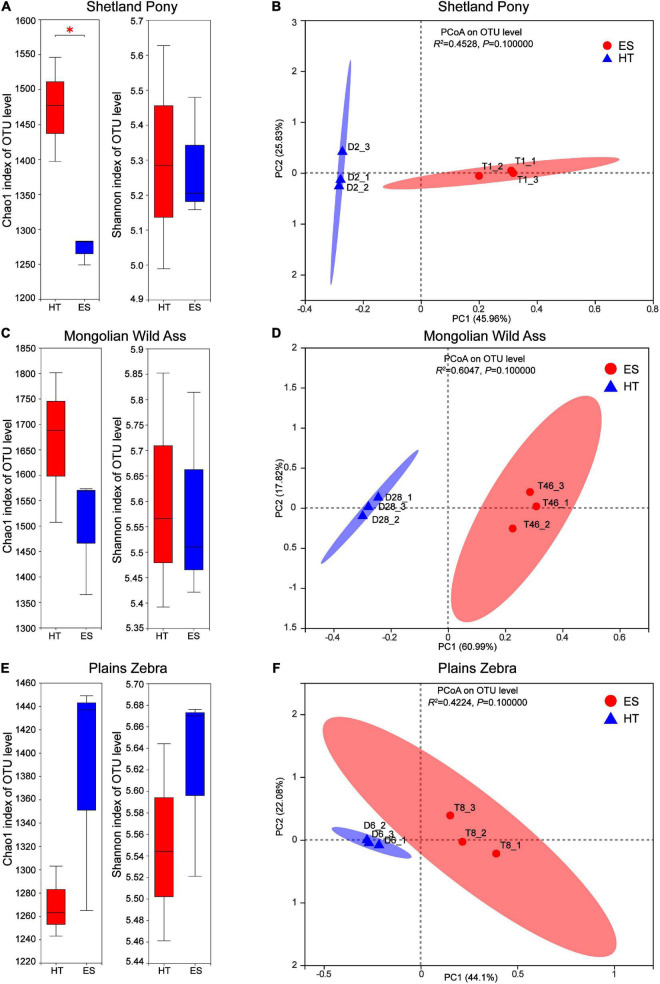
Analysis of intestinal bacterial diversity and community composition of the same in different regions; **(A,B)** are Shetland Pony (SP) intestinal bacterial α-diversity analysis and β-diversity analysis, respectively; **(C,D)** are Mongolian Wild Ass (MA) intestinal bacterialα-diversity analysis and β-diversity analysis, respectively; **(E,F)** are Plain Zebra (PZ) intestinal bacterialα-diversity analysis and β-diversity analysis, respectively. *P < 0.05.

Differences in intestinal bacterial diversity and community composition of the same species in captivity at two wildlife parks (ES and HT) were compared. The results showed that at the OTU level, there was no significant difference (*P* > 0.05) in the bacterial abundance index (Chao1) between MA (Figure 2A) and PZ (Figure 2C), but there was a significant difference (*P* < 0.05) between SP (Figure 2E); the bacterial diversity index (Shannon) was not significantly different (*P* > 0.05) between the same species (Figures 2A,C,E). Meanwhile, the intestinal bacterial community composition (Figures 2B,D,F) could be completely separated between the same species, but none of them were significantly different (*P* > 0.05). Thus, the index of intestinal bacterial diversity between the same species in captivity in these two wildlife parks was highly similar, but there were differences in the composition of intestinal bacterial communities between the same species.

### Analysis of intestinal bacterial diversity among different species

**FIGURE 3:**
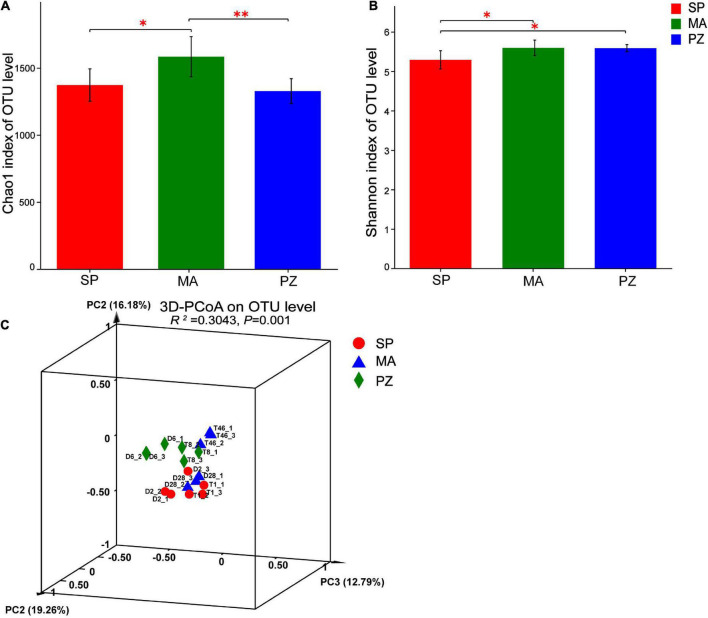
Analysis of intergroup differences in α-diversity and β-diversity of intestinal bacteria in Shetland Pony (SP), Mongolian Wild Ass (MA), and Plain Zebra (PZ); **(A)** α-diversity analysis of intestinal bacteria between groups; **(B)** β-diversity analysis of intestinal bacteria between groups; **(C)** PCoA analysis graph; *P < 0.05 and **P < 0.01.

By analyzing the variability of bacterial abundance index (Chao1) and diversity index (Shannon) between species at OTU level (Figures 3A,B), it is clear that the intestinal bacterial abundance index (Chao1) was not significantly different between SP and PZ (*P* > 0.05), but SP was significantly lower than MA (*P* < 0.05) and PZ was highly significantly lower than MA (*P* < 0.01). The intestinal bacterial diversity index (Shannon) was significantly lower in SP than PZ and MA (*P* < 0.05), but there was no significant difference between PZ and MA (*P* > 0.05). PCoA analysis of bacterial diversity based on the bray_curtis algorithm (Figure 3C) revealed that the differences in intestinal bacterial community composition between species were highly significant (*P* = 0.001). Among them, the degree of explanation in the PC1 dimension was 19.26%, and the composition of the intestinal bacterial community in MA was significantly discrete in the PC1 axis compared to SP and PZ. Thus, we found significant differences in bacterial diversity and community composition among the three equine species.

### Analysis of the species composition of intestinal bacteria among different species

The intestinal bacterial species composition between different species was obtained based on 97% OTU sequence similarity (Supplementary Figure 1). Shetland Pony intestinal bacteria can be clustered 2,158 OTUs, belonging to 289 genera, 147 families and 19 phyla, respectively; MA intestinal bacteria can be clustered 2,321 OTUs, belonging to 321 genera, 153 families and 20 phyla, respectively; PZ intestinal bacteria can be clustered 2,054 OTUs, belonging to 273 genera, 132 families and 19 phyla, respectively. Thus, the proportion of common species of intestinal bacteria in the three equine genera at the four taxonomic levels of OTU, genus, family, and phylum was high, 43.0, 52.6, 60.1, and 85.7%, respectively, and the proportion of bacterial common species increased with increasing taxonomic levels.

### Analysis of intestinal bacterial community composition and species differences among different species

**FIGURE 4:**
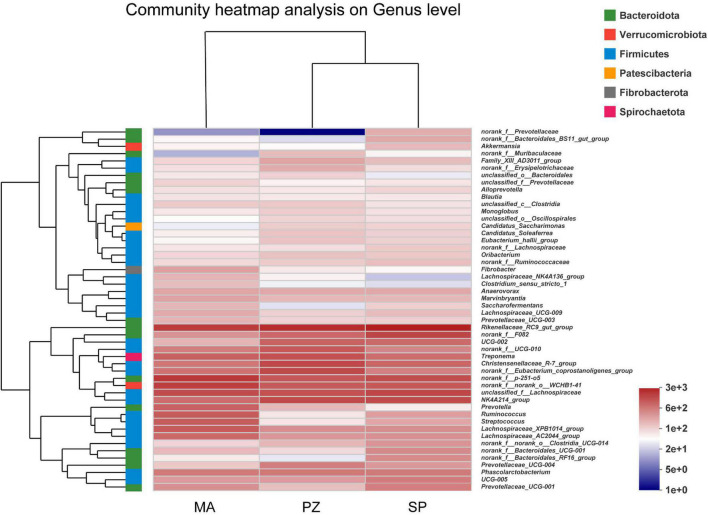
Heatmap of intestinal bacterial community composition.

Analysis of the dominant species and relative abundance of the dominant species at different taxonomic levels of the intestinal bacterial community composition using Bar plots showed that at the phylum level (Supplementary Figure 2A) the main dominant phylum of intestinal bacteria in all three equine species (SP, MA, and PZ) were Firmicutes (51.33, 56.09, and 58.49%) and Bacteroidota (36.56, 28.64, and 27.06%), especially Firmicutes with relative abundances higher than 50% in the intestinal bacteria of all three equine species. In addition to this, Verrucomicrobiota (5.34, 7.41, and 4.56%) and Spirochaetota (3.39, 4.02, and 5.40%) were also the dominant bacterial phylum. Therefore, we found that the dominant phylum species of intestinal bacteria were similar at the phylum level in these three equine species, which is consistent with the results of the analysis of intestinal bacterial species composition. At the family level (Supplementary Figure 2B), the dominant bacterial families among the three equine species were Lachnospiraceae (14.96, 20.87, and 14.04%), Rikenellaceae (12.42, 8.59, and 10.26%), Oscillospiraceae (12.31, 6.12, and 12.08%), Prevotellaceae (6.38, 7.95, and 4.95%), p-251-o5 (5.5, 8.04, and 4.49%), norank_o_WCHB1-41 (4.46, 7.10, and 4.29%), Spirochaetaceae (3.33, 4.00, and 5.40%), Christensenellaceae (3.72, 2.65, and 6.35%). Therefore, we found that the relative abundance of the dominant families of intestinal bacteria at the family level SP and PZ were more similar. At the genus level (Supplementary Figure 2C), the dominant genera in the intestinal bacteria of the three equine species were*Rikenellaceae_RC9_gut_group* (12.20, 7.53, and 9.70%), *norank_f_p-251-o5* (5.50, 8.04, and 4.49%), *unclassified_f_Lachnospiraceae* (6.29, 5.61, and 5.17%), *norank_f_norank_o_WCHB1-41* (4.46, 7.10, and 4.29%), *NK4A214_group* (5.79, 3.16, and 5.80%), and *Christensenellaceae_R-7_group* (3.56, 2.60, and 6.07%). According to the study (Zhao et al., 2016), reported that the dominant phylum of intestinal bacteria in Mongolian and thoroughbred horses were Firmicutes, Bacteroidetes, Spirochaete, etc.; and the dominant genera were *Treponema*, *Ruminococcus*, *Roseburia*, etc. Thus, the present results are very similar to the results of the dominant phylum of intestinal bacteria in Mongolian horses and thoroughbreds, but there are significant differences in the dominant genera at the genus level. Moreover, we found a large number of genera in the intestinal bacteria of these three equine species that have not been clearly classified (Supplementary Figure 2) and need to be studied in depth. Hierarchical clustering analysis of the top 50 bacterial genera in terms of abundance revealed (Figure 4) that these 50 genera were from six bacterial phyla, but these genera were mainly from Fimicutes and Bacteroidota. In addition, the genus composition of the dominant intestinal bacteria in SP and PZ was more similar and differed from that of the dominant MA intestinal bacteria. This result was consistent with the results of the Bar chart analysis of community composition.

**FIGURE 5:**
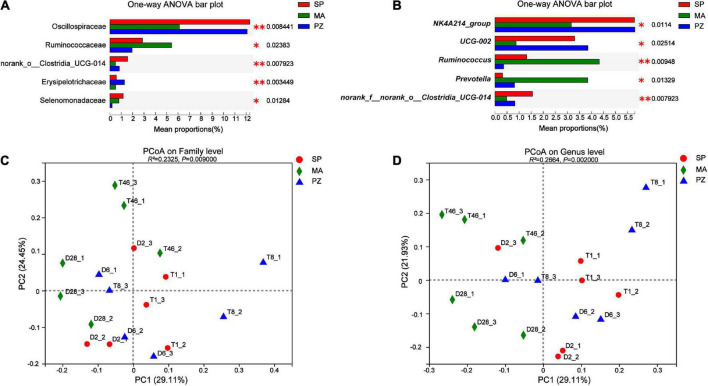
Analysis and significance test for differences in intestinal bacterial species among different species; **(A)** family level; **(B)** genus level; **(C)** family level PCoA plots; **(D)** genus level PCoA plots. *P < 0.05 and **P < 0.01.

Analysis of variance using one-way ANOVA on the composition of the intestinal bacterial community between species and analysis of the top five bacterial colonies with significantly different relative abundances showed that at the phylum level, no significantly different species were found in the intestinal bacteria of the three equine species (*P* > 0.05). This result is in agreement with Park et al. (2021) who compared the intestinal bacterial flora of Jeju and Thoroughbred horses in Korea. At the family level (Figure 5A), the relative abundance of Oscillospiraceae was highly significant higher at SP than at MA (*P* < 0.01) and significantly higher at PZ (*P* < 0.05); the relative abundance of Ruminococcaceae was significantly lower at PZ than at MA (*P* < 0.05); norank_o_Clostridia_UCG-014 relative abundance was significantly higher in SP than PZ (*P* < 0.05) but highly significant than MA (*P* < 0.01); Erysipelotrichaceae relative abundance was highly significant higher in PZ than SP and MA (*P* < 0.01); Selenomonadaceae relative abundance was significantly higher in SP and MA than PZ (*P* < 0.05). At the genus level (Figure 5B), the relative abundance of *NK4A214_group* was highly significant higher in SP than MA (*P* < 0.01) and significantly higher in PZ than MA (*P* < 0.05); The relative abundance of *UCG-002* was significantly higher in SP and PZ than MA (*P* < 0.05); The relative abundance of *Ruminococcus* was significantly higher in MA than SP (*P* < 0.05) but highly significant than PZ (*P* < 0.01) and significantly higher in SP than PZ (*P* < 0.05); *Prevotella* relative abundance was significantly higher in MA than SP and PZ (*P* < 0.05); The relative abundance of *norank_f_norank_o_ Clostridia_UCG-014* was significantly higher in SP than PZ (*P* < 0.05), but highly significant than MA (*P* < 0.01). Thus, we found significant differences in the relative abundance of the three dominant equine species at the family level and the genus level, and this result is consistent with the results of diversity analysis of intestinal bacteria among different species. In addition, PCoA plots were drawn using these five significantly different bacterial families and genera (Figures 5C,D), and it was determined by Adonis analysis that these five families and genera contributed significantly to the differences in the intestinal bacteria of the three equine species.

### Prediction and comparison of gene function of intestinal bacteria among different species

The 16S rRNA genes data of each sample were analyzed by PICRUSt2 software to obtain Pathway level 2 and Pathway level 3 gene functions annotation information. The top five gene functions were annotated in relative abundance at Pathway level 2, and a one-way ANOVA test was used to determine whether there were significant differences in Pathway level 2 gene functions between species (Supplementary Figure 3). The relative abundance of global and overview maps was the highest, followed by carbohydrate metabolism, amino acid metabolism, metabolism of cofactors and vitamins, and energy metabolism in descending order. Among them only the relative abundance of carbohydrate metabolism was significantly lower in PZ than MA (*P* < 0.05). Among the top five gene functions annotated at Pathway level 3, ko01100 (Metabolic pathways) had the highest relative abundance, followed by ko01110 (Biosynthesis of secondary metabolites), ko01230 (Biosynthesis of amino acids), ko01120 (Microbial metabolism in diverse environments), and ko01200 (Carbon metabolism) in descending order of relative abundance. Biosynthesis of amino acids, ko01120 (Microbial metabolism in diverse environments), and ko01200 (Carbon metabolism). Thus, we found high genetic functional similarity in the intestinal bacteria of the three equine species, suggesting that the functional requirements of the equine species for intestinal bacteria are very similar.

## Discussion

The importance of intestinal flora is becoming increasingly known as the relationship between intestinal flora and host is studied in depth (Shi et al., 2022). A large number of microorganisms in the intestinal tract of equine animals digest and absorb the feed they eat, such as high-fiber materials, fats, insoluble proteins, etc. These indigestible substances can be fermented by microorganisms to produce nutrients for the organism (Bulmer et al., 2019). According to studies, research related to the intestinal flora of equine animals is reported to be important for disease prevention (Costa et al., 2015) and promoting growth and development in equine animals (Lindenberg et al., 2019). However, no studies related to the intestinal flora of SP, MA, and PZ have been reported in the study of the intestinal flora of equine animals, so this manuscript used 16S rRNA genes high-throughput sequencing technology to analyze the intestinal bacterial diversity of the above three equine species and to predict the gene function.

We found that the bacterial abundance index (Chao1) of SP was significantly higher than that of HT among the same species, but the bacterial diversity index (Shannon) was not significantly different. Moreover, the bacterial abundance index (Chao1) and diversity index (Shannon) of MA and PZ were not significantly different between ES and HT. Therefore, the effect of feeding conditions on the intestinal bacterial abundance index (Chao1) of MA and PZ was not significant, while the effect on the intestinal bacterial abundance index (Chao1) of SP was significant. According to Kauter et al. (2019) reported that the degree of domestication of the equine genus has an important effect on its intestinal microbial composition. Therefore, we believe that MA and PZ belong to wild animals adapted to wild survival conditions and frequently migrate, so the intestinal bacteria are more resistant to different survival conditions, so the effect of different survival conditions on intestinal bacteria is limited. In contrast, SP has been domesticated for many years and has been kept as pets for a long time, so the range of activity is narrower, so different feeding conditions are likely to have a greater effect on SP intestinal bacteria. Although the intestinal bacteria of the three equine species were completely separated by different regions (ES and HT), there were no significant differences in the composition and absolute abundance of the intestinal bacterial community. The intestinal bacterial abundance index (Chao1) was higher in SP than PZ, but not significantly different between species; significantly lower in SP than MA; and highly significantly lower in PZ than MA, indicating that MA intestinal bacterial abundance was significantly higher than PZ and SP. However, the bacterial diversity index (Shannon) was significantly lower in SP than PZ and MA, while PZ was lower than MA, but not significantly different, thus indicating that PZ had the highest intestinal bacterial homogeneity. In addition, significant differences were found in the composition of the intestinal bacterial communities of different species. According to the study, significant differences were reported in the intestinal bacterial abundance index (Chao1) and diversity index (Shannon) between Korean Jeju horses and thoroughbred horses, in addition to significant differences in the structure of the intestinal bacterial flora between the two species by NMDS analysis (Park et al., 2021). Therefore, we concluded that intestinal bacteria differ significantly among different species in the equine. By Venn diagram, we found that MA intestinal bacteria could cluster 2,321 OTUs, belonging to 321 genera, 153 families and 20 phyla; PZ intestinal bacteria could cluster 2,054 OTUs, belonging to 273 genera, 132 families and 19 phyla; SP intestinal bacteria could cluster 2,158 OTUs, belonging to 289 genera, 147 families and 19 phyla, respectively. In addition, at the phylum level, the three equine genera shared a high percentage of 85.7% of the bacterial phylum. Therefore, we determined that the intestinal bacteria of these three equine species were very similar at the portal level.

In the bacterial community composition Bar, the main common dominant phyla of three equine intestinal bacteria were found to be Firmicutes and Bacteroidota, and the sum of the relative abundance ratios of both phyla was greater than 80%. It was reported that Firmicutes and Bacteroidota were also the dominant bacteria in Tibetan wild asses (Gao et al., 2020), domestic donkeys (Liu et al., 2020), horses (Mshelia et al., 2018). Thus, Firmicutes and Bacteroidota are the core flora of equine intestinal bacteria. Moreover, Firmicutes (Shinkai and Kobayashi, 2007) are the main cellulose degrading bacterial phylum capable of breaking down cellulose into volatile fatty acids, while Bacteroidota (Jiang et al., 2022) are mainly responsible for the degradation of carbohydrates and proteins, and can promote the immune response of the host organism. However, monogastric animals must rely on cellulose degrading bacteria to degrade dietary fiber due to the lack of endogenous cellulose degrading enzymes (Hou et al., 2021). In addition, Verrucomicrobiota and Spirochaetota were also present in the intestinal bacteria of three equine species, with the former relative abundance being metabolically correlated with health status, improving the intestinal barrier and reducing intestinal inflammation (Xiong et al., 2022), and the latter hydrolyzing plant cell walls to produce complex polysaccharides, complex vitamin B, and also having potential to degrade proteins (Hernandez et al., 2022). At the family level, Lachnospiracea is the dominant family among three species of equine intestinal bacteria that are present in the intestine of most healthy humans and are a potentially beneficial bacterium involved in the metabolism of a wide range of carbohydrates, with fermentation leading to the production of acetate and butyric acid providing the main source of energy for the host (Wang et al., 2015; Vacca et al., 2020). In addition, some shared dominant families related to cellulose, starch and pectin digestion, such as Rikenellaceae and Prevotellaceae, and shared dominant Oscillospiraceae related to systemic inflammation and altered intestinal permeability were also identified (Terzo et al., 2020). The results of this study provide directions for targeted screening of specific functional probiotics, and we can obtain target strains by selective media for the dominant bacteria and use them in probiotic studies. At the genus level, *Rikenellaceae_RC9_gut_group* had the highest relative abundance, and we found a large number of genera in the intestinal bacteria of equids that are not yet clearly classified and need to be studied in depth. Most of the genera with higher relative abundance from the intestinal bacterial Heatmap were found to be from Fimicutes and Bacteroidota. Furthermore, the genus composition of the dominant intestinal bacteria in SP and PZ was more similar and differed from that of the dominant MA intestinal bacteria. The analysis of relative abundance differences revealed that no species were found to be significantly different at the phylum level; however, at the family level, species with significant differences in relative abundance between SP and PZ were found to be norank_o__Clostridia_UCG-014, Erysipelotrichaceae and Selenomonadaceae; Species with significant differences in relative abundance between SP and MA were Oscillospiraceae and norank_o__Clostridia_UCG-014; species with significant differences between PZ and MA were Oscillospiraceae, Ruminococcaceae, Erysipelotrichaceae, and Selenomonadaceae. At the genus level, species with significant differences in relative abundance between SP and PZ were *Ruminococcus* and n*orank_f__norank_o__Clostridia_UCG-014*; Species with significant differences in relative abundance between SP and MA were *NK4A214_group, UCG-002*, *Ruminococcus*, *Prevotella* and *norank_f__norank_o__Clostridia_UCG-014*; species with significant differences between PZ and *MA were NK4A214_group*, *UCG-002*, *Ruminococcus*, and *Prevotella*. In addition we found a high number of species with significant differences in relative abundance between SP and MA and between PZ and MA at the genus level, but a low number of species with significant differences in relative abundance between SP and PZ. Moreover, it is interesting to note that this result corroborates with the results of the interspecies PCoA analysis, indicating that the intestinal bacterial community composition is more similar between SP and PZ compared to MA. In conclusion, there were no significant differences in the relative abundance of species among the three equine species at the phylum level, but species differences arose mainly between the family level and the genus level.

The intestine as a complex ecosystem is closely related to the immune level, inflammatory response and glucose homeostasis of the organism (Adak and Khan, 2019). Gene functions analysis is an important component of intestinal bacterial research and an important method for studying how microbes regulate host metabolism (Ogata et al., 1999). Prediction of gene function by PICRUSt2 in the intestinal bacteria of three equine species revealed that the top five metabolism-related genes in Pathway level 2 were global and overview maps, carbohydrate metabolism, amino acid metabolism, metabolism of cofactors and vitamins, and energy metabolism, in order of relative abundance. The top five metabolism-related genes in Pathway level 3 in terms of relative functional abundance were metabolic pathways, biosynthesis of secondary metabolites, biosynthesis of amino acids, microbial metabolism in diverse environments and carbon metabolism. In addition, the top five pathways in Pathway level 2 and Pathway level 3 in terms of relative abundance of related gene functions all belonged to metabolism, suggesting that metabolism function is very strong in the intestinal bacterial gene functions of equids, which is consistent with previous studies (Meale et al., 2017). We found that the relative abundance of carbohydrate metabolism in MA was higher than that in SP and PZ because the relative abundance of *Ruminococcus* and *Prevotella* in MA intestinal bacteria was significantly higher than that in SP and PZ, as both genera are closely associated with carbohydrate metabolism (Tremaroli and Backhed, 2012). In summary, studying the diversity of intestinal bacteria and their gene functions in equine genera is important for the exploitation of intestinal microorganisms.

## Conclusion

With the increasing research on intestinal flora, researchers have found an inextricable relationship between intestinal flora and organism health. Therefore, intestinal flora can be a good predictor of the health status of animals. In addition, species composition is the most important aspect of intestinal flora studies. Thus, this study analyzed the species composition and gene functions prediction of the intestinal flora of three equine species using high-throughput sequencing of 16S rRNA genes. It was found that the bacterial abundance index (Chao1) and diversity index (Shannon) and PCoA analysis of three species of equine animals in captivity in two wildlife parks (ES and HT) revealed that only the intestinal bacterial abundance index (Chao1) of SP was significantly different between the same species (*P* < 0.05), while the rest of the indexes were not significantly different (*P* > 0.05), but there were significant differences between, however, there were significant differences between species (*P* < 0.05). From the Venn diagram analysis, it was found that MA intestinal bacteria could cluster 2,321 OTUs, belonging to 321 genera, 153 families, and 20 phyla; PZ intestinal bacteria could cluster 2,054 OTUs, belonging to 273 genera, 132 families, and 19 phyla; SP intestinal bacteria could cluster 2,158 OTUs, belonging to 289 genera, 147 families, and 19 phyla, respectively, phylum. Moreover, the proportions of bacterial shared species in the three equine genera were high at four taxonomic levels of OTU, genus, family, and phylum (43.0, 52.6, 60.1, and 85.7%, respectively). The dominant phyla among the three equine animal intestinal bacteria were Firmicutes and Bacteroidota, of which the family with the highest relative abundance was Lachnospiraceae and the genus with the highest relative abundance was *Rikenellaceae_RC9_gut_group*. ANOVA in the relative abundance of species revealed no significantly different species at the phylum level (*P* > 0.05); while a large number of species with differences were found at the family and genus levels, especially four significantly different species between PZ and MA at both levels. In addition, we found that the main contribution to significant differences in the relative abundance of species differing at the genus level came between SP and MA and between PZ and MA. The highest functional relative abundance of metabolism-related genes at Pathway level 2 was global and overview maps; the highest functional relative abundance of metabolism-related genes at Pathway level 3 was biosynthesis of secondary metabolites. In sum, the intestinal bacterial community composition and diversity of the three equine species in captivity in the two wildlife parks were significantly different, but their metabolism-related genes were functionally similar. Moreover, the results of this manuscript fill the gap in the study of intestinal bacterial diversity in SP, MA, and PZ, which provides directions for the targeted screening of probiotics with specific functions.

## Data availability statement

The datasets presented in this study can be found in online repositories. The names of the repository/repositories and accession number(s) can be found in the article/Supplementary material.

## Ethics statement

The animal study was reviewed by the Experimental Animal Welfare and Ethics Committee of Inner Mongolia Agricultural University, conformed to the ethical principles and agreed to the implementation of the project.

## Author contributions

WB: conceptualization and writing–review and editing. WB and JY: formal analysis, writing, and original draft preparation. JY and XY: investigation. JY and YH: data curation. WB, JY, YH, ML, and XY: methodology, took responsibility for all aspects of their work, and approved the submitted version.

## Footnotes

1
https://github.com/OpenGene/fastp

2
http://www.cbcb.umd.edu/software/flash

3
http://www.drive5.com/uparse/

4
https://www.arb-silva.de//

5
http://www.genome.jp/kegg/
