# Supplementary material for: The diversity analysis and gene function prediction of intestinal bacteria in three equine species
Source: Front Microbiol. 2022 Sep 7;13:973828. doi: 10.3389/fmicb.2022.973828 (PMC9490377; doi:10.3389/fmicb.2022.973828)
Supplement: Supplementary file 1 [file Image_1.pdf]

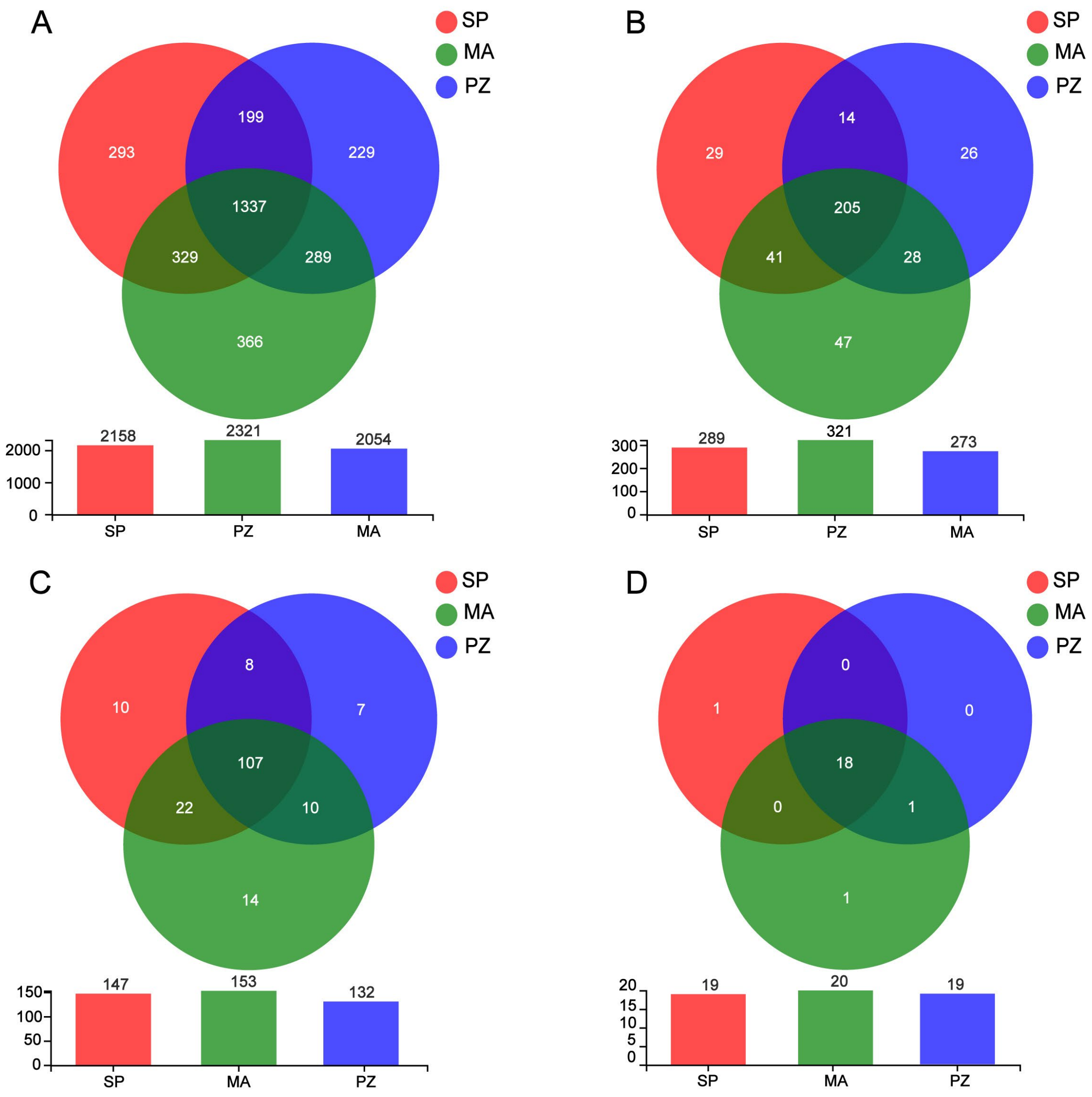


**Fig.S1** Venn diagram of species composition of bacteria at different taxonomic levels; A, OTU level; B, genus level; C, family level; D, phylum level.
