# Supplementary material for: The diversity analysis and gene function prediction of intestinal bacteria in three equine species
Source: Front Microbiol. 2022 Sep 7;13:973828. doi: 10.3389/fmicb.2022.973828 (PMC9490377; doi:10.3389/fmicb.2022.973828)
Supplement: Supplementary file 2 [file Image_2.pdf]

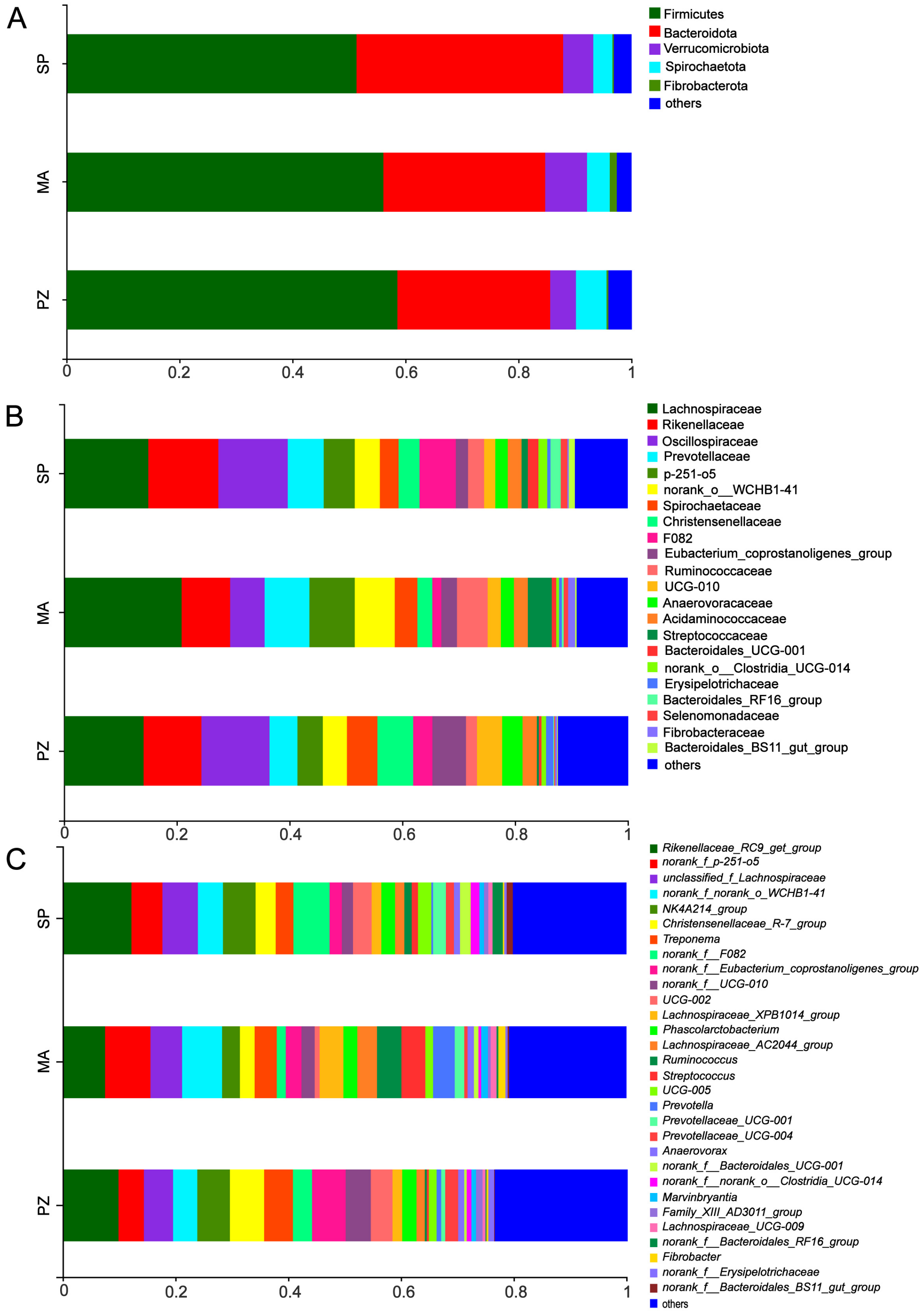

Fig.S2 Bar diagram of community composition of bacteria at different taxonomic levels; A, phylum level; B, family level; C, genus level.
