# Supplementary material for: The diversity analysis and gene function prediction of intestinal bacteria in three equine species
Source: Front Microbiol. 2022 Sep 7;13:973828. doi: 10.3389/fmicb.2022.973828 (PMC9490377; doi:10.3389/fmicb.2022.973828)
Supplement: Supplementary file 3 [file Image_3.pdf]

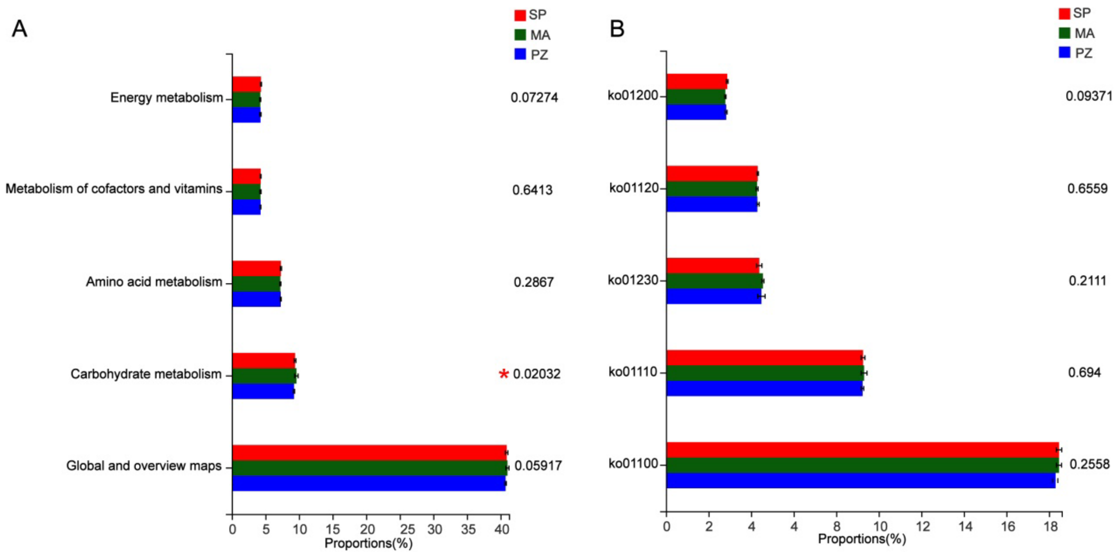

Fig.S3 Relative abundance distribution of genes related to metabolic pathways in the intestinal flora of three equine species; A, Pathway level 2; B, Pathway level 3. * represents P<0.05 and ** represents P<0.01.
